# Natraemia variations induced by acute dialysis in critically ill patients: a database study

**DOI:** 10.1038/s41598-022-18897-z

**Published:** 2022-09-02

**Authors:** Gilles Troché, Virginie Laurent, Alexis Ferré, Gwenaelle Jacq, Marine Paul, Sybille Merceron, Stephane Legriel

**Affiliations:** Service de Réanimation Médico-Chirurgicale, Hôpital André Mignot, Centre Hospitalier de Versailles, 177 rue de Versailles, 78150 Le Chesnay, France

**Keywords:** Biophysical chemistry, Kidney diseases, Metabolic disorders

## Abstract

Natraemia is often abnormal in critically ill patients and may change rapidly during renal replacement therapy (RRT). This database study in a single intensive care unit (ICU) evaluated natraemia before and after the first RRT session for acute kidney injury. Of 252 patients who required RRT in 2018–2020, 215 were included. Prevalences were 53.9% for hyponatraemia (≤ 135 mmol/L) and 3.7% for hypernatraemia (> 145 mmol/L). Dialysate sodium was ≥ 145 mmol/L in 83% of patients. Median dialysis sodium gradient was 12 mmol/L, with a value above 16 mmol/L in 25% of patients. Median natraemia increased from 135 before to 140 mmol/L after RRT, the median hourly increase being faster than recommended, at 1.0 mmol/L [0.2–1.7]. By multivariate analysis, the only variable significantly associated with the RRT-induced natraemia change was the dialysis sodium gradient [odds ratio, 1.66; 95% confidence interval 1.39–2.10]. Pearson’s correlation coefficient between the gradient and the natraemia change was 0.57. When performing RRT in ICU patients, in addition to the haemodynamic considerations put forward in recommendations, the dialysis sodium gradient deserves careful attention in order to control natraemia variations. Studies to devise a formula for predicting natraemia variations might prove helpful to confirm our results.

## Introduction

Natraemia is often abnormal in critically ill patients and may change rapidly during renal replacement therapy (RRT). Hyponatraemia, defined as a serum sodium concentration (SNa^+^) below 135 mmol/L, is common in ICU admitted patients, notably those with critical illnesses. Reported incidences range from 15 to 30%^1–3^. Risk factors include age older than 70 years; kidney, heart, or liver failure; hypovolaemia; recent administration of intravenous fluids or of diuretics; weight gain or undernutrition; and hypoalbuminaemia^1,2,4–6^. The causes of hyponatraemia are manifold but can be categorised as hypovolaemic, euvolaemic, or hypervolaemic and as hypotonic, isotonic, or hypertonic. Urine output and the clinical setting assist in the diagnosis^2,7^. Together with glucose and urea, sodium is a major plasma osmole, and correcting hyponatraemia is particularly important when glycaemia and/or uraemia are elevated^8^. Hypernatraemia, i.e., serum sodium > 145 mmol/L, is less common.

Both hyponatraemia and hypernatraemia are associated with increased morbidity rates and stay lengths^3,5^. Moreover, SNa^+^ values < 126 or > 165 mmol/L are associated with increased mortality^4^. Plasma hypo-osmolarity can cause neurological complications^9^, and lower natraemia has been reported to correlate with higher adjusted mortality^6,7^. When seeking to correct hyponatraemia, a slow increase is recommended to avoid neurological complications, notably osmotic demyelination syndrome^1^.

Thus, correcting dysnatraemia and obtaining this correction at the appropriate rate are crucial. However, the best method for predicting SNa^+^ variations induced by dialysis is unclear^6,7,10^. The effect of dialysis on SNa^+^ depends in part on the dialysate sodium concentration, about which no clear recommendations exist^3,6,7,10^. In our practice, a standard concentration is often used, without adjustment during RRT, according to SNa^+^ or clinical findings. In ICU patients with acute kidney injury (AKI) requiring RRT, no specific strategy for correcting abnormal SNa^+^ values exists, and SNa^+^ may vary considerably during RRT, potentially affecting patient outcomes^3,6,11–13^.

The primary objective of this retrospective study of a prospectively established, single-centre database was to describe the SNa^+^ change during the first RRT session in ICU patients. The secondary objective was to identify factors associated with the SNa^+^ change, knowledge of which might help to determine the optimal RRT parameters for each patient.

## Methods

The study database was reported to the French data protection authority (*Commission Nationale de l’Informatique et des Libertés*, #220969, on 24 November 2018). The data were anonymised before use for the study. French law does not require written informed consent for retrospective analyses of anonymised healthcare data (Journal Officiel de la République Française n°0160 on 13 July 2018). However, all patients or families were informed of the study, in writing, at the time of prospective data collection; those who were unwilling to participate were not included. This study protocol was approved by the ethics committee of the *Société de Réanimation de Langue Française* (#CE-SRLF 20-74 on 29 September 2020) and registered on the French national healthcare database (*Institut National des Données de Santé*). All methods were performed in accordance with the relevant guidelines and regulations.

### Study design

We conducted a retrospective observational study of information collected prospectively at our ICU in a university hospital in France between 1 January 2018 and 29 February 2020.

### Patients

Consecutive patients who required acute RRT in our ICU during the study period were eligible. We did not include patients with uncontrollable haemodynamic instability, death during the first RRT or patients on chronic RRT.

Our 20-bed ICU has a 2.5/1 nurse/patient ratio. The dialysers were three 5008 CorDiax machines (Fresenius Medical Care, Bad Homburg, Germany) and one Diacap^®^ Pro 13H machine (B. Braun Medical, Melsungen, Germany). The dialysate was SW 376 A (K^+^, 1 mmol/L) or SW 381 A (K^+^, 3 mmol/L), both from B. Braun Medical. The dilution volume was 1 + 34 and concentrations in the final solution were Na^+^, 138.0 mmol/L; Ca^++^, 1.5 mmol/L; Mg^++^, 0.5 mmol/L; Cl^−^, 110 mmol/L; HCO_3_^−^, 32.0 mmol/L; acetate, 3 mmol/L; and glucose, 5.5 mmol/L. A written protocol is available, but the bedside physician chose the dialysis parameters [duration; Na^+^ (127-151 mmol/L), K^+^ (1 or 3 mmol/L), and HCO_3_^−^ (24–40 mmol/L) concentrations; ultrafiltration use and flow; blood flow; and dialysate flow]. Anticoagulation and catecholamine use were also at the discretion of the bedside physician.

### Data collection

For each patient, we extracted the study data from our electronic ICU database. Baseline data were age, sex, height, weight, Simplified Acute Physiology Score II (SAPSII) at ICU admission, and main diagnosis. We also recorded the type and duration of ventilatory assistance, if used, and the type and dosage of catecholamines if used. Regarding RRT, we collected the duration; dialysate Na^+^, K^+^, and HCO_3_^−^ concentrations; total ultrafiltration volume if relevant; blood flow, dialysate flow, and total dialysed blood volume; anticoagulant and catecholamine use; and Kt/V as a measure of dialysis efficacy (K, clearance; t, time; and V, body water volume). Serum values before and after RRT were collected for Na^+^, K^+^, HCO_3_^−^, urea, and creatinine. Body mass index (BMI) was computed as weight (kg) divided by height^2^ (in m), using weights measured before and after RRT.

The dialysis sodium gradient was computed by subtracting the pre-RRT SNa^+^ from the dialysate sodium concentration. The RRT-induced SNa^+^ change was obtained by subtracting pre-RRT SNa^+^ from post-RRT SNa^+^. Effective plasma osmolarity was computed as [natraemia (mmol/L) × 2] + [glycaemia (mmol/L)/3.3] + [uraemia (mmol/L)/17.5]^8^ and the osmolarity change by subtracting pre-RRT osmolarity from post-RRT osmolarity.

### Statistical methods

Quantitative variables were described as median [interquartile range] and qualitative variables as number (percentage). The prevalence of hyponatraemia and hypernatraemia before RRT were computed using cut-offs of 135 and 145 mmol/L, respectively. RRT-induced SNa^+^ changes were classified as abnormal if < 0 or > 10 mmol/L. We compared categorical variables using the χ^2^ test and continuous variables using Student’s *t* test. Correlations were assessed by computing Pearson’s correlation coefficient. In the figures, the results are shown as datapoint clouds and correlation lines.

We used logistic regression to determine whether variables listed in Table 5 were associated with RRT-induced natraemia variations. Non-collinear variables that yielded *p* values smaller than 0.05 by univariate analysis or were clinically relevant were considered for entry into a multivariable model. The Hosmer–Lemeshow goodness-of-fit test and area under the receiver operating characteristics curve (AUC-ROC) estimated by the C-statistic were computed for the final models. Associations of factors with RRT-induced SNa^+^ changes are reported as odds ratios (ORs) with their 95% confidence intervals (95%CIs). Missing data were ignored.

All tests were two sided, and *p* values < 0.05 were considered significant.

Analyses were performed using R statistical software version 4.0.0 (R Foundation for Statistical Computing, Vienna, Austria; http://www.R-project.org. Accessed June 15, 2022).

## Results

### Patients

**Figure 1:**
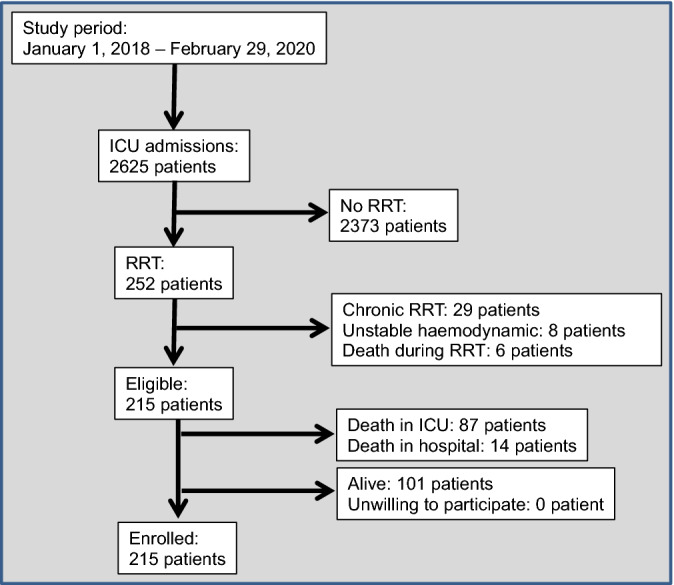
Patients flow chart.

Figure 1 is the patient flow chart. Of 2625 patients admitted to our ICU during the study period, 252 (9.6%) required RRT. Among them, 37 were excluded because they had chronic kidney disease, uncontrollable haemodynamic instability or died during the first RRT session.

Table 1 reports the main features of the 215 included patients. Among them, 87 (40.5%) were admitted from a ward in our hospital and the remaining 128 (59.5%) from home via the emergency department. Only 35 (16.3%) patients were post-operative. Most patients (n = 154, 71.6%) required invasive mechanical ventilation during the ICU stay. There are not missing data except for proteins (before = 79.1%; after = 88.0%), albumin (before = 39.5%; after = 52.3%) and blood gases (before = 11.6%; after = 15.7%).

**Table 1:** Main characteristics of the 215 study patients and first renal replacement therapy (RRT) session.

Variables	N (%) or Median [interquartile range]
**Patient characteristics at ICU admission**	
Females	77 (35.8)
Age (years)	69.0 [61.0–76.5]
BMI (kg/m^2^)	28.0 [24.7–32.0]
ICU stay length (days)	7.0 [2.0–16.0]
SAPS II	59 [46–77]
**RRT characteristics**	
Time from ICU admission to RRT (days)	0 [0–1]
Blood flow (mL/min)	180 [150–200]
Dialysate flow (mL/min)	500 [500–500]
Dialysis duration (h)	4.0 [4.0–6.0]
Dialysate sodium (mmol/L)	145.0 [145.0–150.0]
Dialysate glucose (mmol/L)^a^	5.0
Dialysate urea (mmol/L)^a^	0.0
Ultrafiltration	61 (28.4)
Total ultrafiltration volume (mL) (n = 61 patients)	0 [0–700]
Dialysis duration (h)	4.0 [4.0–6.0]
Kt/V	1.0 [0.7–1.5]
Total dialysed blood volume (L)	47.4 [36.7–61.6]
Total ultrafiltration (mL)	0 [0–700]

*RRT* renal replacement therapy, *BMI* body mass index, *ICU* intensive care unit, *SAPS II* Simplified Acute Physiological Score II.

^a^The concentrations of glucose and urea in the dialysate were identical in all patients.

### Renal replacement therapy (RRT)

Table 1 reports the main RRT characteristics. The dialysate sodium concentration was chosen initially then left unchanged throughout the RRT session in all patients; it was 150 mmol/L in 99 (46.0%) patients, 145 mmol/L in 80 (37.2%) patients, 140 mmol/L in 24 (11.2%) patients, 135 mmol/L in 5 (2.3%), other in 7 patients.

Table 2 shows findings before and after the first RRT session. Of the 215 patients, before RRT, 116 (53.9%) had SNa^+^ ≤ 135 mmol/L and 8 (3.7%) > 145 mmol/L. The lowest value was 121 mmol/L and the highest 150 mmol/L. No patient had known severe dyslipidaemia or para-proteinaemia. Neither glycerol nor mannitol was used in any of the study patients. The dialysis sodium gradient resulted in a median SNa^+^ increase of 1 mmol/L/h [0.2–1.7] during the RRT session. After RRT, 158 (73.5%) patients had SNa^+^ values within the normal range, compared to 91 (42.4%) before RRT.

**Table 2:** Characteristics before and after the first renal replacement therapy (RRT) session.

N = 215 patients	N (%) or Median [interquartile range]		
	Before 1st RRT	After 1st RRT	*p* value
**Clinical characteristics**			
Weight (kg)	80.0 [69.1–92.6]	85.0 [71.1–97.3]	0.12
Temperature (°C)	36.6 [35.9–37.3]	36.8 [36.2–37.3]	< 0.001
MAP (mmHg)	74 [67–84]	79 [68–87]	0.03
HR (bpm)	92 [73–113]	94 [79–113]	0.11
RR (bpm)	22 [19–27]	22.0 [19–27]	0.74
SpO_2_ (%)	97 [95–99]	97 [95–99]	0.82
Urine output (mL/kg/h)	0.11 [0.00–0.68]	0.06 [0.00–0.36]	0.08
Mechanical ventilation	132 (61.4)	135 (62.8)	0.05
Catecholamine infusion	124 (57.6)	129 (60.0)	0.03
**Biological characteristics**			
Sodium (mmol/L)	135 [131–139]	140 [137–143]	< 0.001
Glycaemia (mmol/l)	7.8 [6.0–11.7]	6.4 [5.3–8.2]	< 0.001
Blood urea (mmol/L)	19.7 [12.2–31.8]	8.2 [5.1–15.2]	< 0.001
Calculated osmolarity (mmol/L)	274 [266–282]	283 [276–288]	< 0.001
Potassium (mmol/L)	4.8 [4.1–5.6]	4.1 [3.7–4.5]	< 0.001
Bicarbonate (mmol/L)	17.4 [14.5–21.4]	22.5. [19.4–25.4]	< 0.001
Protein (g/L)	58.0 [52.0–64.0]	57.5 [53.0–63.8]	0.86
Albumin (g/L)	24.5 [19.0–29.0]	25.0 [19.0–28.5]	0.93
Haemoglobin (g/dL)	10.0 [8.4–12.2]	9.9 [8.3–11.8]	0.002
Creatinine (μmol/L)	257 [162–513]	141 [93–301]	< 0.001
pH	7.24 [7.16–7.34]	7.36 [7.26–7.43]	< 0.001
Lactate (mmol/L)	2.4 [1.0–7.0]	2.3 [1.2–6.1]	0.02
Dialysate-to-serum Na^+^ gradient (mmol/L)	12.0 [8.0–16.0]	7.0 [4.5–10.0]	< 0.001
∆Na^+^ (mmol/L)	–	5.0 [1.0–7.5]	–
∆Osmolarity (mmol/L)	–	8.1 [0.7–13.8]	–

*RRT* renal replacement therapy, *MAP* mean arterial pressure, *HR* heart rate, *RR* respiratory rate, ∆: change during RRT (value after RRT − value before RRT).

### Complications and mortality

Overall, 87 patients died before ICU discharge and 14 before hospital discharge. Table 3 reports the ICU and hospital mortality rates according to SNa^+^ before and after the first RRT session. In patients with hypernatraemia before RRT, no additional deaths occurred in the hospital after ICU discharge, whereas of the 15 patients with hypernatraemia after RRT, 6 (40%) died on the wards. Of the 116 patients with hyponatraemia before RRT, 14 (12%) died after ICU discharge to the wards; of the 42 with hyponatraemia after RRT, 6 (14%) died after ICU discharge to the wards.

**Table 3:** ICU and hospital mortality according to serum sodium concentration (SNa^+^) before and after the first renal replacement therapy (RRT) session.

Serum sodium levels (mmol/L)	Before 1st RRT N (%)	ICU mortality N (%)	Hospital mortality N (%)	After 1st RRT N (%)	ICU mortality N (%)	Hospital mortality N (%)
**Hyponatremia:** Na^+^ ≤ 135	116 (53.9)	43 (37.0)	57 (48.3)	42 (19.5)	13 (30.2)	19 (45.2)
Mild: 130 ≤ Na^+^ ≤ 135	80	33 (41.2)	39 (47.5)	37	12 (32.4)	16 (43.2)
Moderate: 125 ≤ Na^+^ ≤ 129	26	10 (38.5)	11 (42.3)	4	1 (25.0)	2 (50.0)
Severe : Na^+^ < 125	10	0	7 (70.0)	1	0	1 (100)
**Hypernatremia:** Na^+^ > 145	8 (3.7)	3 (37.5)	3 (37.5)	15 (7.0)	0	6 (40.0)
Severe: Na^+^ > 155	0	0	0	0	0	0
**Normal sodium level: **135 < Na^+^ ≤ 145	91 (42.3)	41 (45.1)	41 (45.1)	158 (73.5)	74 (46.8)	76 (48.1)

*ICU* intensive care unit, *RRT* renal replacement therapy.

Table 4 compares ICU and hospital mortality across groups defined by the magnitude of the SNa^+^ change induced by RRT. Overall, mortality increased with the magnitude of the SNa^+^ increase. However, some sub-groups were small. Both ICU and hospital mortality were very high.

**Table 4:** ICU and hospital mortality according to the change in natraemia induced by the first renal replacement therapy (RRT) session.

Natraemia change (mmol/L)	N of patients	ICU mortality N (%)	Hospital mortality N (%)
< − 5.0	3	0 (0)	0 (0)
− 5.0 to 0.0	43	16 (37.2)	19 (44.1)
0.1–5.0	82	33 (40.2)	39 (47.6)
5.1–10.0	66	30 (45.4)	32 (48.5)
10.0–15.0	18	6 (33.3)	9 (50.0)
≥ 15.1	3	2 (66.7)	2 (66.7)
Total	215	87 (40.5)	101 (47.0)

*ICU* intensive care unit, *RRT* renal replacement therapy.

### Variables associated with serum sodium (SN^+^) changes during renal replacement therapy (RRT)

Tables 5 and 6 report the variables significantly associated with the RRT-induced natraemia change by univariate and multivariate analysis, respectively. By multivariate analysis, only the dialysis sodium gradient before RRT predicted the SNa^+^ change induced by RRT.

**Table 5:** Variables associated with the natraemia change by univariate analysis.

Variable	OR (95% CI)	*p* value
Age	0.98 (0.95–1.01)	0.11
Male	0.31 (0.12–0.78)	0.013
Medical condition	0.42 (0.15–1.18)	0.10
Urine output before RRT	1.44 (0.95–2.19)	0.085
Natraemia before RRT	0.77 (0.69–0.85)	< 0.0001
Natraemia before RRT < 135 mmol/L	30.4 (4.00–231.1)	0.001
Kalaemia before RRT	1.32 (0.95–1.84)	0.10
Bicarbonate before RRT	0.89 (0.81–0.96)	0.005
Urea before RRT	1.03 (1.00–1.06)	0.037
Albumin before RRT	0.95 (0.87–1.03)	0.21
Haemoglobin before RRT	0.87 (0.71–1.06)	0.17
Creatinine before RRT	1.00 (1.00–1.00)	0.48
Calculated osmolarity before RRT	0.88 (0.83–0.92)	< 0.0001
Dialysate sodium	1.12 (0.98–1.29)	0.11
Dialysate potassium	0.95 (0.67–1.34)	0.77
Dialysate bicarbonate	1.09 (0.93–1.28)	0.29
RRT duration	1.19 (0.94–1.50)	0.15
Total dialysed blood volume	1.01 (1.00–1.03)	0.096
Final Kt/V	1.57 (0.47–5.20)	0.46
Dialysis-sodium gradient	1.69 (1.38–2.06)	< 0.0001

*OR* odds ratio, *95% CI* 95% confidence interval, *RRT* renal replacement therapy.

**Table 6:** Variables associated with the natraemia change by multivariate analysis.

Variable	OR (95% CI)	*p* value
Bicarbonate before RRT	0.91 (0.81–1.02)	0.11
Urea before RRT	1.01 (0.98–1.05)	0.41
Dialysis sodium gradient	1.66 (1.39–2.10)	< 0.001

Hosmer–Lemeshow: 0.95; AUC-ROC: 0.94.

### Correlation analysis

**Figure 2:**
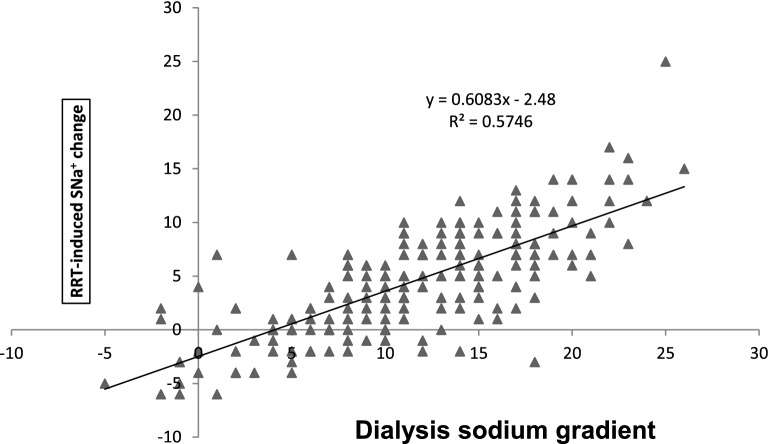
Correlation between the dialysate sodium gradient and the RRT-induced change in serum sodium (Pearson’s r^2^, 0.76).

**Figure 3:**
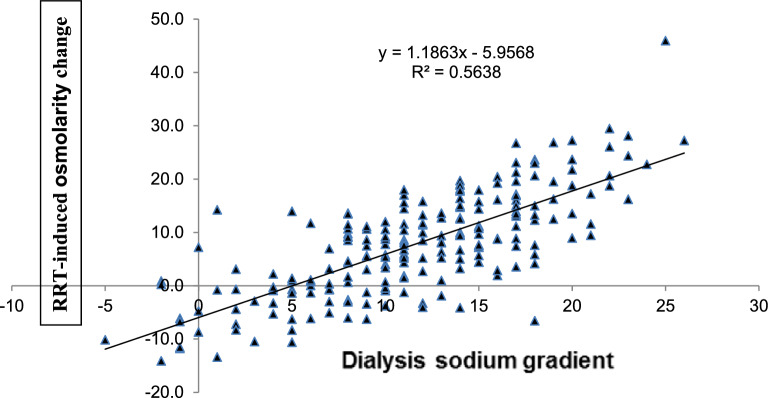
Correlation between the dialysate sodium gradient and the RRT-induced change in serum osmolarity (Pearson’s r^2^, 0.75).

Pearson’s coefficient (r^2^) computed to assess the correlation between the dialysis sodium gradient and the RRT-induced SNa^+^ change was 0.57 (Fig. 2). The SNa^+^ change was predicted by the following formula: (0.6083 × dialysate-blood sodium gradient in mmol/L) − 2.48. For the correlation between the dialysis sodium gradient and the RRT-induced change in blood osmolarity, r^2^ was 0.56 (Fig. 3). The blood osmolarity change was predicted by the formula: (1.1863 × dialysate-blood sodium gradient in mmol/L) − 5.9568.

## Discussion

Our single-centre retrospective review of prospectively collected data showed that the first RRT session in patients with severe critical illness increased SNa^+^ by a median of 5 mmol/L, from 135 to 140 mmol/L. This increase was ascribable to the dialysis sodium gradient and to the decreases in glycaemia and urea induced by RRT. In patients with hyponatraemia, the rate of SNa^+^ correction was faster than recommended, at 1 mmol/L. The increase in calculated osmolarity was acceptable. By multivariate analysis, only the dialysis sodium gradient before RRT independently predicted the SNa^+^ change induced by RRT.

Mortality was high in our cohort. The high SAPSII score and large proportions of patients who required mechanical ventilation and/or catecholamines indicated considerable disease severity. Mortality increased with the magnitude of the RRT-induced SNa^+^ change but the differences were not statistically significant, possibly due to the small size of the sub-groups.

The optimal rate of SNa^+^ correction is controversial and has not been assessed in randomised controlled trials^14–16^. Also, no consensus exists about adjusting the sodium dialysate concentration to SNa^+^^7,17,18^. In patients with chronic hyponatraemia (> 48 h), slow correction over 2–3 days at a rate no higher than 6–8 mmol/L/day is recommended to prevent neurological complications^2,5,9^. Rapid correction carries a risk of permanent brain damage, notably osmotic demyelination syndrome^2,4,5,9^. Since the 1990s, the recommended rate of hyponatraemia correction has fallen from 0.5–1.0 to 0.25–0.5 mmol/L/h^9^. Nonetheless, in patients with hyponatraemia and severe neurological complications, SNa^+^ should be increased rapidly by 4–6 mmol/L^9^. However, neurological outcomes also depend on serum, urea, glucose, and potassium levels and on comorbidities such as chronic alcohol abuse, undernutrition, and advanced liver failure^9^. High urea levels such as those in our cohort may protect the brain against the adverse effects of rapid SNa^+^ variations^2^. In our study, the median rate of RRT-induced SNa^+^ change was + 1.0 mmol/L/h. In 21 patients, SNa^+^ increased by more than 10 mmol/L. Thus, in ICU patients, choice of Na dialysate concentration should not only depend on haemodynamis stability but also on initial natraemia and osmolarity. Given the severity of the critical illness, high mortality, and limited data on baseline neurological status, we were unable to determine whether this large SNa increase was associated with poorer neurological outcomes. SNa^+^ decreased during RRT in 28 patients but the variation was moderate and the lowest post-RRT value was 124 mmol/L. Osmolarity decreased during RRT in nearly a fourth of the patients but, again, the change was moderate and therefore unlikely to have had an adverse impact. Glucose and urea play major roles in osmolarity, and SNa^+^ therefore does not always correlate with osmolarity^9^.

The need to achieve or maintain haemodynamic stability is a key consideration when choosing the dialysate sodium concentration and dialysis sodium gradient for acute RRT^6,16^. AKI in ICU patients is often accompanied with other organ failures including shock requiring vasopressor therapy. A positive dialysis sodium gradient increases natraemia, volaemia, and arterial blood pressure while decreasing intracellular and interstitial volumes. To improve the haemodynamic stability of critically ill patients requiring acute RRT, a dialysate sodium concentration value of 145 mmol/L is recommended^6^. In our study, the proportion of patients receiving catecholamines was not significantly different before and after RRT.

The RRT-induced SNa^+^ change depends not only on the dialysate sodium concentration and dialysis sodium gradient but also on blood flow rate, dialysate flow rate, and changes in serum glucose and urea levels. We estimated effective plasma osmolarity as [SN^+^ (mmol/L) × 2] + [glycaemia (mmol/L)/3.3] + [uraemia (mmol/L)/17.5]^8^. Plasma conductivity is a surrogate for plasma osmolarity and also depends on SNa^+^, glucose, and urea. Algorithms can be embedded in dialyser monitors to tailor the dialysate sodium concentration to each patient’s needs in order to maintain conductivity unchanged^11^. In our study, conductivity was monitored to detect rapid changes, but changes in conductivity were not used to modify the dialysate sodium concentration.

Hypernatraemia was rare in our cohort and was twice as common after than before RRT. In a database study, the rate of hypernatremia correction was not associated with higher frequencies of death, seizures, or cerebral oedema^14^.

A major limitation of our study is the retrospective design. However, the proportion of missing data was substantial only for serum protein and albumin. Given the single-centre recruitment, the results may not apply to all ICUs. No standardised acute RRT protocol was available, and treatment heterogeneity may therefore have occurred across intensivists. We did not measure osmolarity but instead obtained an estimate by applying Worthley’s equation, although no consensus exists about the best estimation method^8^. Most patients started RRT within hours after ICU admission and we were therefore unable to separate chronic from acute hyponatraemia. As most patients had severe acute illness requiring sedation, mechanical ventilation, and catecholamine therapy, specific neurological manifestations of dysnatraemia could not be assessed.

## Conclusion

Over half our cohort of ICU patients requiring RRT had hyponatraemia. This proportion is probably an underestimation, given the high serum glucose and urea levels. Nonetheless, pre-RRT osmolarity was low. The high dialysis sodium gradient resulted in a median 5-mmol/L natraemia increase, a median SNa^+^ increase rate of 1 mmol/L/h, and nearly a tenth of patients having an SNa^+^ increase greater than 10 mmol/L. These findings are in contradiction to current recommendations. The dialysis sodium gradient was the only variable independently associated with the SNa^+^ change in our cohort. A positive correlation was observed between dialysis sodium gradient and SNa change. Thus in clinical practice, choice of sodium dialysate concentration depends on the initial natraemia and the final natraemia objective.

## Data Availability

Readily reproducible materials described in the manuscript, including all relevant raw data, will be freely available to any scientist wishing to use them for non-commercial purposes. Reasonable requests for access to the clinical study data can be submitted via email to the corresponding author.

## Abbreviations

AKI: Acute kidney injury
BMI: Body mass index
CNIL: *Commission Nationale de l’Informatique et des Libertés* (French data protection authority)
HR: Heart rate
ICU: Intensive care unit
INDS: *Institut National des Données de Santé* (French healthcare data institute)
IQ: Interquartile
MAP: Mean arterial pressure
RR: Respiratory rate
RRT: Renal replacement therapy
SAPS II: Simplified Acute Physiological Score II
SNa: Serum sodium concentration
SRLF: *Société de Réanimation de Langue Française* (French-speaking critical-care society)

## References

[1] Spasovski G (2014). Clinical practice guideline on diagnosis and treatment of hyponatraemia. Eur. J. Endocrinol..

[2] Hoorn EJ, Zietse R (2017). Diagnosis and treatment of hyponatremia: Compilation of the guidelines. J. Am. Soc. Nephrol..

[3] Pirklbauer M (2020). Hemodialysis treatment in patients with severe electrolyte disorders: Management of hyperkalemia and hyponatremia. Hemodial. Int..

[4] Joergensen D, Tazmini K, Jacobsen D (2019). Acute dysnatremias—A dangerous and overlooked clinical problem. Scand. J. Trauma. Resusc. Emerg. Med..

[5] Weismann D, Schneider A, Höybye C (2016). Clinical aspects of symptomatic hyponatremia. Endocr. Connect..

[6] Hecking M (2012). Predialysis serum sodium level, dialysate sodium, and mortality in maintenance hemodialysis patients: The dialysis outcomes and practice patterns study (DOPPS). Am. J. Kidney Dis..

[7] Rhee CM, Ayus JC, Kalantar-Zadeh K (2019). Hyponatremia in the dialysis population. Kidney Int. Rep..

[8] Rasouli M (2016). Basic concepts and practical equations on osmolality: Biochemical approach. Clin. Biochem..

[9] Verbalis JG (2013). Diagnosis, evaluation, and treatment of hyponatremia: Expert panel recommendations. Am. J. Med..

[10] Brunet P *et al*. Mise au point sur le dialysat utilisé en hémodialyse. https://www.sfndt.org/sites/www.sfndt.org/files/medias/documents/mise_au_point_sur_le_dialysat_utilise_en_hemodialyse_0.pdf. Accessed 21 July 2022.

[11] Ságová M, Wojke R, Maierhofer A, Gross M, Canaud B, Gauly A (2019). Automated individualization of dialysate sodium concentration reduces intradialytic plasma sodium changes in hemodialysis. Artif. Organs.

[12] Geng X, Shi E, Wang S, Song Y (2020). The efficacy and safety of low dialysate sodium levels for patients with maintenance hemodialysis: A systematic review and meta-analysis. Int. J. Sur..

[13] Yessayan L, Yee J, Frinak S, Szamosfalvi B (2016). Continuous renal replacement therapy for the management of acid-base and electrolyte imbalances in acute kidney injury. Adv. Chronic Kidney Dis..

[14] Chauhan K (2019). Rate of correction of hypernatremia and health outcomes in critically ill patients. Clin. J. Am. Soc. Nephrol..

[15] Sterns RH (2018). Treatment of severe hyponatremia. Clin. J. Am. Soc. Nephrol..

[16] Munoz Mendoza J, Arramreddy R, Schiller B (2015). Dialysate sodium: Choosing the optimal hemodialysis bath. Am. J. Kidney Dis..

[17] Hecking M (2012). Dialysate sodium concentration and the association with interdialytic weight gain, hospitalization and mortality. Clin. J. Am. Soc. Nephrol..

[18] Boyle R (2019). Managing hyponatremia. JAAPA..

